# Cytoprotective and anti-inflammatory effects of PAL31 overexpression in glial cells

**DOI:** 10.1186/1423-0127-21-60

**Published:** 2014-07-17

**Authors:** Fan-Wei Tseng, Dann-Ying Liou, May-Jywan Tsai, Wen-Cheng Huang, Henrich Cheng

**Affiliations:** 1 Department and Institute of Pharmacology, School of Medicine, National Yang-Ming University, Taipei, Taiwan; 2 Neural Regeneration Laboratory, Neurological Institute, Taipei Veterans General Hospital, No 322, Shih-Pai Road, Sec. 2, Taipei 112, Taiwan; 3 Center for Neural Regeneration, Neurological Institute, Taipei Veterans General Hospital, Taipei, Taiwan; 4 Faculty of Medicine, School of Medicine, National Yang-Ming University, Taipei, Taiwan

**Keywords:** Spinal cord injury, Proliferation related acidic leucine-rich protein, Inflammation, H_2_O_2_ toxicity, Astroglia, C6 glioma, Microglia

## Abstract

**Background:**

Acute spinal cord injury (SCI) leads to a series of reactive changes and causes severe neurological deficits. A pronounced inflammation contributes to secondary pathology after SCI. Astroglia respond to SCI by proliferating, migrating, and altering phenotype. The impact of reactive gliosis on the pathogenesis of SCI is not fully understood. Our previous study has identified an inflammatory modulating protein, proliferation related acidic leucine-rich protein (PAL31) which is upregulated in the microglia/macrophage of injured cords. Because PAL31 participates in cell cycle progression and reactive astroglia often appears in the injured cord, we aim to examine whether PAL31 is involved in glial modulation after injury.

**Results:**

Enhanced PAL31 expression was shown not only in microglia/macrophages but also in spinal astroglia after SCI. Cell culture study reveal that overexpression of PAL31 in mixed glial cells or in C6 astroglia significantly reduced LPS/IFNγ stimulation. Further, enhanced PAL31 expression in C6 astroglia protected cells from H_2_O_2_ toxicity; however, this did not affect its proliferative activity. The inhibiting effect of PAL31 on LPS/IFNγ stimulation was observed in glia or C6 after co-culture with neuronal cells. The results demonstrated that the overexpressed PAL31 in glial cells protected neuronal damages through inhibiting NF-kB signaling and iNOS.

**Conclusions:**

Our data suggest that PAL31upregulation might be beneficial after spinal cord injury. Reactive gliosis might become a good target for future therapeutic interventions.

## Background

Spinal cord injury (SCI) causes severe and often permanent neurological deficits through direct trauma and delayed secondary damage [1,2]. The cascade of secondary injury is mediated by inflammatory response, involving macrophage and microglial activation [3-5]. Neurons in injured cord have become more sensitive to a secondary injury. Several lines of evidence have reported that attenuating inflammation decreases secondary damage and the functional deficit after SCI [6,7]. Astrocytes respond to SCI by proliferating, migrating, and altering phenotype. Reactive astrocytes can be found within the lesion, and they can also constitute a physical barrier between the lesion and the surrounding tissue [8,9]. These cells are considered to be detrimental for axonal regeneration, but their function remains elusive.

After SCI, intracellular Ca^2+^ is transiently increased and initiates several damaging effects. One of these effects is activation of nitric oxide synthase (NOS) in mitochondria and cytoplasm. The activated NOS can generate peroxynitrite anion (ONOO^-^), a reactive product of superoxide radical with nitric oxide. Peroxynitrite can trigger cellular damage by a variety of mechanisms [10,11]. Glial cells (astrocytes and microglia) synthesize NO after the transcriptional expression of inducible NOS which can be promoted by the endotoxin lipopolysaccharide (LPS) and/or by certain cytokines, such as interferon-γ (IFNγ) [12,13].

Cyclic AMP has been shown to play critical role in neural regeneration [14] and this effect is mediated through the activation of protein kinase A (PKA). Previously, we had identified a phospho-motif in PKA substrates that correlated with SCI rat neural regeneration [15]. This novel molecule was identified as a proliferation related acidic leucine-rich protein (PAL31) with molecular weight of 31 kDa. PAL31 had been discovered in the developing nervous system and its expression level decreased following maturation. The functions of PAL31 has been discovered that participated in cell cycle progression [16], caspase-3 inhibition [17], and inflammatory modulation [15]. Interestingly, PAL31 was found to express in the infiltrated macrophages in the epicenter of the injured spinal cord and the amount of PAL31 reached its peak level over 6 days after transection of spinal cord [15]. This suggests that PAL31 may play a modulatory role on inflammation during SCI. On the other hand, PAL31 had been reported to co-localize with PCNA which participated in DNA replication and repair [16]. Proliferation of astroglia and microglia is increased at 1–7 days after SCI [18]. Astrocytes in spared gray and white matter of spinal cord undergo hypertrophy or gliosis after injury. Because PAL31 participates in cell cycle progression and reactive astroglia often appears in the injured cord, PAL31 could be expressed in spinal glia after SCI. In the present study, we aim to examine whether PAL31 is involved in glial modulation after spinal cord injury. To reveal the molecular mechanism of PAL31 in astroglia, a full-length PAL31 cDNA was first prepared. We conducted PAL31 overexpressing experiments in primary cultures derived from spinal cord tissues to mimic the interaction of glia and neurons *in vivo*. Here we present evidence showing that enhanced expression of PAL31 in astroglia might be beneficial after spinal cord injury.

## Methods

### Materials

Lipopolysaccharide (LPS; *Escherichia coli* O111:B4) and antibiotics were purchased from Sigma-Aldrich (St. Louis, USA). Rat interferon-γ (IFNγ) was from Peprotech. A methylthiazol tetrazolium (MTT) kit was from Chemicon (USA). Culture multi-wells and pipettes were obtained from Orange Scientific (Graignette, Belgium). Cultured media and fetal bovine serum (FBS) were purchased from Gibco (Invitrogen Corporation, USA). Other reagents were purchased from Sigma-Aldrich unless stated otherwise.

### Spinal cord injury

Adult female Spraque-Dawley (SD) rats (250 ± 20 g) were used for inducing spinal cord injury (SCI) and the operation procedures have been described elsewhere [19-22]. Briefly, adult female SD rats were anesthetized with isoflurane and a laminectomy was performed. Vertebrate thoracic T7–T10 was exposed. Rats underwent a complete ‘transection’ operation at thoracic T8. Following injury, the incision was closed and sutured. Each rat was then returned to its cage. To avoid urinary tract infections, manual emptying of the urinary bladder was carried out twice daily. All surgical interventions and animal care were performed in accordance with the Laboratory Animal Welfare Act, the Guide for the Care and Use of Laboratory Animals, National Institutes of Health and were approved by the Animal Committee of Taipei Veterans General Hospital, Taiwan.

### Cell culture

Mixed neuronal/glial cultures were prepared from embryonic SD rat spinal cords at gestation days 14–16 as described previously [23-25]. Briefly, cells were dissociated with mixtures of papain/protease/deoxyribonuclease I (0.1:0.1: 0.03%) and plated in poly-lysine coated multiwell plates (12 × 24 mm) or on mixed glial cultures (for co-culture study, see below). Mixed glial cultures were prepared from adult SD rat spinal cords following methods described previously [26,27] with modifications. Briefly, spinal cords, free of meninges, were dissociated by trypsinization. The dissociated cells were passed through nylon cloths (70 um), plated in 75 cm^2^ flasks and maintained in DMEM supplemented with 10% FBS. The cells were incubated at 37°C in a water-saturated atmosphere of 5% CO_2_/95% air. Confluent cultures were purified on the 10th day by shaking 5 hrs at 180 rpm to remove the suspended cells. Cultures in the flasks were replated into multiwell plates. Subconfluent mixed glial cell cultures were used for PAL31 or GFP overexpression experiment (see below). C6 glioma were obtained from ATCC (Manassas, VA, USA Catalog No. 30–2004). The base medium for this cell line is ATCC-formulated F-12 K Medium. C6 cultures were maintained in ATCC-formulated F-12 K Medium supplemented with 2.5% FBS and 12.5% horse serum during cell expansion. C6 glioma were adapted to DMEM + 10% FBS one passage before cDNA transfection experiments and thereafter [28].

### Construction of PAL31 vector and transfection of vector to cultures

Vectors used in these studies were constructed by inserting the human PAL31 cDNA into the pEGFP-C1 vector (BD Biosciences Clontech, San Jose, CA, USA). Briefly, full-length human PAL31cDNA was amplified from Marathon-Ready cDNA (Clontech, CA, USA) by PCR. Primers for PAL31 were PAL31-forward 5’-GAA,TTC,AAT,GGA,CAT,GAA,GAG, GAG-3’and PAL31-reverse 5’-GAA, GGA,GAA,GAT,GAC,TAA,TCT,AGA-3’. Full length PAL31 cDNA was then ligated into the EcoRI and XbaI site of pEGFP-C1 vector yielding the plasmid EP, and verified by DNA sequencing. For cell culture transfection, 5 μg each of the recombinant plasmid EP (pPAL31) or EV (pEGFP-C1) were first mixed with T-Profect transfection reagent (JF Ji-Feng Biotechnology, Taiwan) in cultured medium containing 5% FBS. The resulted mixtures were added to cultures and incubated for 16 hr. The cells were then replaced with growth medium. The resulted transfected cells were used in the present study.

### Construction of the pal31 siRNA

We designed oligonucleotides as pal31 gene hairpin siRNA to knockdown endogenous PAL31 expression in cultures. Two sequences of siRNA were custom-ordered synthesis from Ambion Life Technologies as shown in Additional file 1 supporting information. siRNA was transfected to C6 cultures using a Lipofectamine RNAiMax reagent (Invitrogen) by a protocol suggested by the provider (166 pmol siRNA for cells in 6 cm dish). Two ~ three days after confirming the knockdown of PAL31 in C6 cells, the cultures were exposed to H_2_O_2_ challenge.

### H_2_O_2_ or LPS/IFNγ exposure

GFP or PAL31-overexpressing cells were maintained in 10% FCS containing medium. Doses of hydrogen peroxide (0.5-1 mM) were added to the culture medium for 4 hr. The survived cells were analyzed for the degree of MTT reduction. In the experiments of LPS/IFNγ exposure, transduced cells were maintained in serum-containing medium (DMEM +10% FBS). During LPS /IFNγ treatment, the culture medium was switched to DMEM +4% FBS. LPS (4 μg/mL) and IFNγ (10 ng/mL) were added to cultures and incubated for 2 hrs ~ 2 days. The medium was then saved for nitrite assay, while cells were fixed for immunostaining or harvested for western blot analysis.

### Fluorescent immunohistochemistry of spinal cord tissue

Tissue slides were washed twice with PBS, permeabilized with 0.5% Triton X-100 in PBS for 5 min, and incubated in 0.05% Tween 20 in PBS for 5 min at room temperature. Fixed tissue were pre-incubated for 30 min in PBS containing 2% BSA and then incubated for 1 h at 37°C with goat anti-PAL31 (Novus, NB100-1199), ED-1 (Serotec, MCA341) and GFAP (Chemicon, AB5040) (dilution 1:500~1:1000). After three washes with PBS, they were incubated for 30 min with FITC-conjugated anti-goat antibody (dilution 1:100; Zymed, CA) and Alexa fluro 568 (Molecular probes, A11004). The tissues were incubated in 50 mg/ml of DAPI in PBS for nuclei staining, and were washed twice with PBS before mounting with artifading reagent. Images of samples were obtained with a fluorescent microscope equipped with fluorescence optics and a CCD camera. Micrography was performed using a 10X and 20X objective and images were processed with imaging software (MetaMorph Imaging System, Universal Imaging Corp, Downingtown, PA, US).

### Cell survival MTT assay

Degree of MTT reduction, which is reduced to a blue formazan product by viable cells, followed a method described in Tsai et al. [26]. Following treatment, aliquots of MTT stock solution (5 mg/ml) was added to each well and incubated at 37°C for 3 hr. At the end of incubation, the media were aspirated and cells were solubilized in acid isopropanol (0.04-0.1 N HCl in isopropanol). The resulted solution was measured spectrophotometrically at 590 nm with background subtraction at 630–690 nm.

### The nitrite assay

The production of nitric oxide (NO) was assayed as the accumulation of nitrite in the medium using colorimetric reaction with Griess reagents (1% sulfanilaminde/0.1% naphthylethylene diamine dihydrochloride/2% phosphoric acid) as described by Tsai et al. [23] and Tseng et al. [29]. After LPS/IFNγ treatment, the culture medium was collected, mixed with Griess reagents and incubated at room temperature for 10 min. The absorbance of the resultant products was measured at 540 nm. Sodium nitrite (NaNO_2_) was used as the standard to calculate nitrogen dioxide (NO_2_) concentrations.

### Western blot analysis

At designated time, cells were homogenized in lysis buffer containing 10 mM Tris, pH 7.4, 50 mM NaCl, 1% NP-40, 1× protease inhibitor (Roche, Indianapolis, IN, USA), 30 mM Na_4_P_2_O_7_, 1 mM Na_3_VO_4_, and 30 mM NaF. Protein concentration was assayed using a Bio-Rad DC kit (Hercules, CA, USA). The protein extract (20 μg/lane) was next separated on 12.5% sodium dodecyl sulfate–polyacrylamide gel electrophoresis (SDS–PAGE) and then transferred to a polyvinylidene difluoride filter (Millipore, Bedford, MA, USA) as described [30]. Primary antibodies such as Anp32b (PAL31) (Novus, NB100-1199), PCNA (Santa cruz, SC-9857), GFP (Santa cruz, SC-5385), iNOS (Abcam, 47350), pNF-kB (Cell signaling, #3033), NF-kB (Cell signaling, #8242) and actin (Santa cruz, SC-1616), were used at 1000–2000 fold dilution. Horseradish peroxidase-conjugated antibody was used as the secondary antibody (Santa Cruz Biotechnology, Santa Cruz, CA, USA). The protein bands were visualized by enhanced chemiluminescence development (PerkinElmer Life Sciences, Waltham, MA, USA). The detailed procedures for the western blotting were the same as described in the New England Nuclear western blot manual, which is provided by the manufacturer.

### Statistical analysis

**Figure 1:**
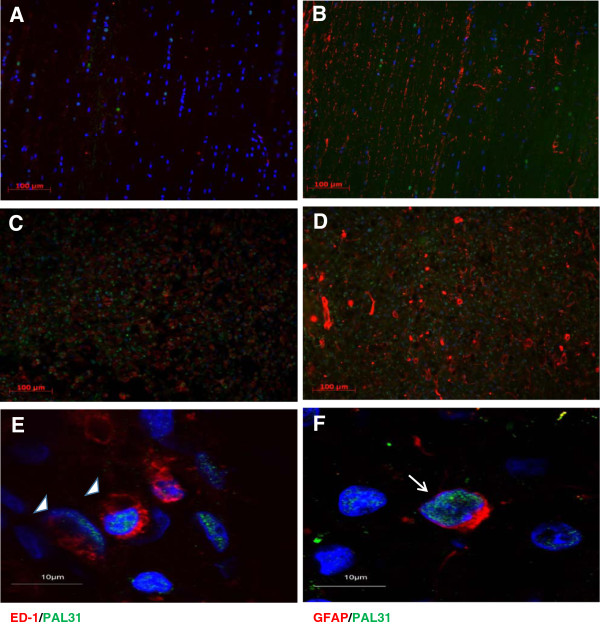
**Upregulated PAL31 expression was observed not only in microglia/ macrophage but also in glial cells near the injury site. (A)** Normal thoracic spinal cord section showing few ED-1 positive cells (red). **(B)** Normal thoracic spinal cord section showing GFAP positive cells (red). **(C)** Representative micrograph showing colocalization of upregulated PAL31 (green) with microglia/macrophage (ED1 positive cells) **(D)** Representative micrograph showing colocalization of upregulated PAL31 with astroglia (GFAP positive cells). **(E)** High power image showing the colocalization of upregulated PAL31 with ED1 positive macrophage/microglia (arrowhead) **(F)** High power image demonstrating the colocalization of upregulated PAL31 with GFAP positive astroglia (arrow). Magnification: 200X **(A-D)** and 1000X **(E&F)**.

**Figure 2:**
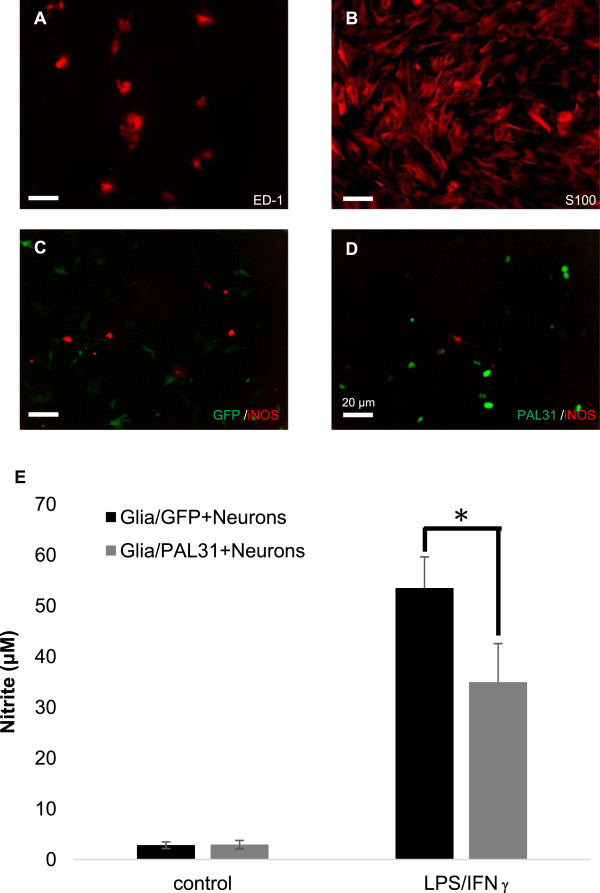
**Overexpression of PAL31 in mixed glial culture effectively reduced LPS/IFNγ stimulation after co-culture with spinal cord neuron-glial cultures. (A)** ED-1- immunoreactive microglia/macrophage in confluent mixed glial cultures **(B)** S100- immunoreactive astroglia in confluent mixed glial cultures **(C)** LPS/IFNγ-stimulated neuron-glial co-cultures (Glia/GFP + neuron) showing expression of both iNOS and GFP **(D)** LPS/IFNγ-stimulated neuron-glial co-cultures (Glia/PAL31+ neuron) showing expression of both iNOS and PAL31. **(E)** Quantitative analysis of released nitric oxide, as a form of nitrite, in LPS/IFNγ-stimulated or non-stimulated neuron-glial co-culture. Mixed glial cultures were transfected with pGFP or pPAL31 before seeding spinal cord neurons. Co-cultures were then treated with LPS/IFNγ for 2 days. Medium were saved for nitrite level determination, while cells were processed for immunostaining with iNOS. *P < 0.05, n = 4. PAL31 overexpressing co-cultures (+LPS/IFNγ) compared to GFP overexpressing co-cultures (+LPS/IFNγ). Magnification 200X **(A-D)**.

All measurements were performed blind to each group. Experimental data were expressed as the mean of independent values ± s.e. and were analyzed using one-way analysis of variance (ANOVA) followed by Bonferroni post hoc test. Data in Figure 1D and Figure 2B was analyzed using two-way ANOVA and Bonferroni *post hoc* test. P values less than 0.05 were considered statistically significant.

## Results

### Upregulated PAL31 expression was observed not only in microglia/macrophage but also in glia cells near the injury site

In our previous study, PAL31 was found to express in the infiltrated macrophages in the epicenter of the injured spinal cord [15]. The amount of PAL31 reached its peak level over 6 days after transection of spinal cord. The present work employed same injury model, collected thoracic spinal cords at one week post-injury and examined whether PAL31 expression was present in other cell types of this injured spinal cord.As expected, PAL31 upregulation was observed in ED-1-immunoreactive microglia/macrophage (Figure 1C and E; Arrowheads), compared to that in normal cord (Figure 1A). Interestingly, near the transected stump, GFAP-positive astroglia were found to express PAL31 (Figure 1D and F). As shown in Figure 1F (Arrow), PAL31 immunoreactivity (green) co-localized with GFAP-positive cells (red). This phenomenon indicated that PAL31 might have important roles in glia cell after spinal cord injury.

### Anti-inflammatory effect of overexpressing PAL31 in primary mixed glia culture

To evaluate the effects of upregulated PAL31 in glial cells, we constructed full-length PAL31 cDNA and conducted PAL31 overexpressing experiments in primary cultures derived from spinal cord tissues to mimic the interaction of glia and neurons *in vivo*. To identify cell types in mixed glial cultures prepared from adult spinal cords, we used glial cell markers anti-ED-1 [31,32] and anti-S100 [33] for immunocytochemistry. As shown in Figure 2A and B, mixed glial cultures comprised predominant astroglia (S100-immunoreactive cells) and few activated ED-1-immunoreactive microglia. Human full-length PAL31 cDNA was successfully made and further inserted into pEGFP-C1 yielding a plasmid with GFP-tagged PAL31. The resulted plasmid each, pEGFP or pPAL31, was added to mixed glial cultures with transfection reagent (T-Profect). As a result, more than 50% of cells expressed GFP. GFP expression was found in the cytosol of Glia/GFP-cultures (Figure 2C, green) and in the nucleus of PAL31/GFP-cultures (Figure 2D, green), indicating correct subcellular localization of GFP-tagged PAL31. Spinal cord neuronal cultures were then seeded to GFP- or PAL31-transfected mixed glial cultures (Glia/GFP or Glia/PAL31) for co-cultures and examined whether PAL31 overexpression conferred protection against stimulation by LPS and interferon-γ (IFNγ). LPS, an endotoxin, is a powerful immune challenge associated with an increase of numerous cytokines and inducible nitric oxide synthase (iNOS) expression. Figure 2C and D shows that numbers of LPS/IFNγ-stimulated iNOS positive cells were less in Glia/PAL31-cocultures than in Glia/GFP-cocultures. The co-cultured media were subjected to nitrite assay to evaluate nitric oxide production. Figure 2E showed the released nitrite was 53.49 ± 6.15 μM in Glia/GFP + Neurons cocultures stimulated by LPS/IFNγ. The Glia/PAL31 + Neurons + LPS/IFNγ released significantly less nitrite (34.94 ± 7.61 μM; p < 0.05). Thus, overexpressed PAL31 in glia not only attenuated LPS/IFNγ-stimulated iNOS expression but also reduced the accompanied NO production. To further examine the functions of PAL31 in glial cells, we used homogenous C6 astroglioma cell line to substitute the primary mixed glia.

### Overexpressing PAL31 in C6 cell did not affect the cell proliferation

**Figure 3:**
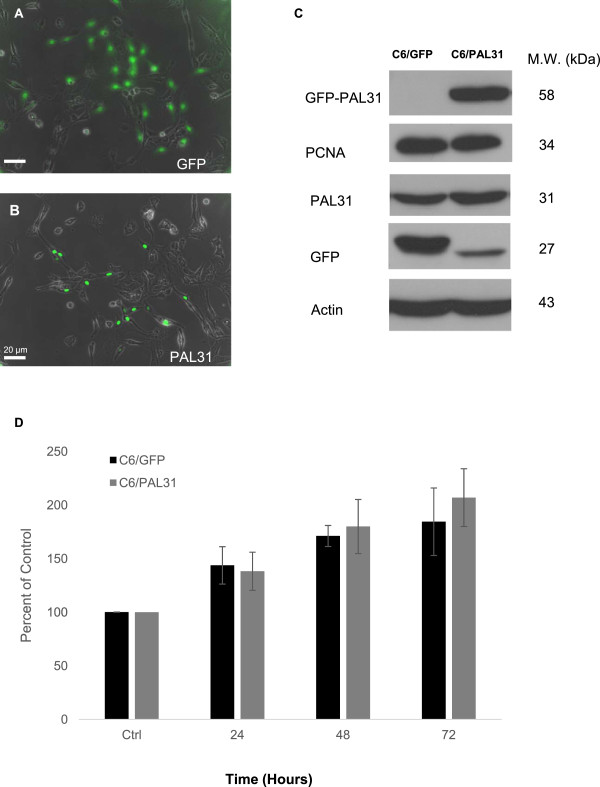
**Overexpression of PAL31 in C6 did not affect the cell proliferative activity. (A)** GFP/bright field-superimposed micrograph in GFP overexpressing cells showing > 50% transfective efficiency **(B)** GFP-PAL31/bright field-micrograph in PAL31 overexpressing C6 showing correct nuclear localization of GFP-tagged PAL31 and transfection effeciency. **(C)** Western blot analysis showing the expression of GFP (~27 kDa), GFP-tagged PAL31 (~58 kDa), PCNA (~34 kDa), endogenous PAL31 (~31 kDa) and Actin (~43 kDa) in GFP- or PAL31-C6 cultures. The level of PCNA, a protein expressing in nuclear during DNA synthesis, in cells did not alter after overexpression of GFP or GFP-tagged PAL31. Actin works as a loading control. **(D)** MTT assay in the transfected cells at 4 different time intervals (from Ctrl to 72 hours). Ctrl represents the cells after subculture and overnight incubation. The data in each time points were analyzed by two-way ANOVA and Bonferroni post hoc test. No significance, compared GFP and GFP tagged PAL31 groups. n = 3. Magnification 100X **(A-B)**.

C6 cells were transfected with pEGFP or pGFP-PAL31 using T-profect. The transfection efficiency reached >50% (GFP/phase-contrast superimposed micrograph; Figure 3A and B). GFP-tagged PAL31 was found to express in the nucleus of C6 (Figure 3B), whereas GFP expression was predominant in the cytosol of GFP-C6 (Figure 3A). Western blot analysis of these cultures further confirmed the corrected size of overexpressed PAL31 in C6/PAL31 (~58 kDa for GFP-tagged PAL31; Figure 3C).The antibody ANP32b could detect endogenous PAL31 protein (~31 kDa) and the GFP antibody detected the tag of the transfected PAL31 and GFP protein. The results of western blot showed that the overexpressed PAL31 cell lysate could be detected by PAL31 and GFP antibodies at ~58 kDa and by anti-GFP at ~27 kDa. This indicated correct size of GFP tagged PAL31 with some breakdown signal (~27 kDa). This did not affect the results of our experiment. Endogenous PAL31 was detected by ANP32b at 31 kDa with equivalent level in both C6/GFP and C6/PAL31 cells (Figure 3C). Levels of actin were used as a loading control. PAL31 had been reported to co-localize with PCNA which participated in DNA replication and repair [16]. Therefore, we examined whether PAL31 overexpressing in C6 affected PCNA expression and the cell’s proliferative activity (by MTT assay). As a result, enhanced PAL31 expression in cells did not affect the expression level of PCNA (Figure 3C) as well as the MTT assay results (Figure 3D). The proliferative activity (degree of MTT reduction) in C6 at all time intervals (0 ~ 72 hr) were not significantly different between C6/GFP and C6/PAL31 groups (Figure 3D).

### Enhanced PAL31 expression in C6 protected cells from H_2_O_2_ toxicity

**Figure 4:**
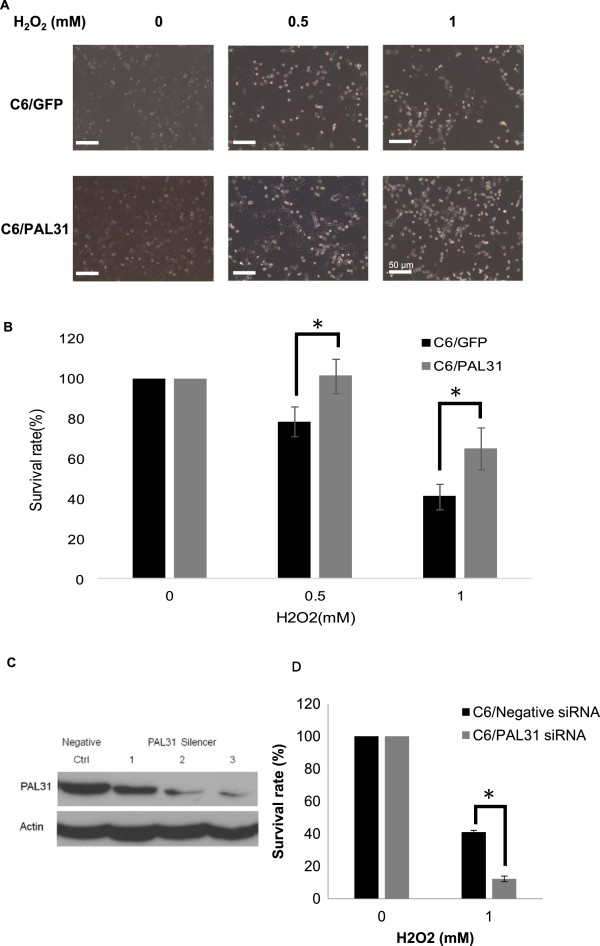
**Effect of overexpression or knockdown of PAL31 in C6 on H**_**2**_**O**_**2**_**-induced toxicity. (A)** Representative micrographs showing C6 overexpressing GFP or GFP-tagged PAL31 after being treated with H_2_O_2_ (0 ~ 1 mM) for 4 hours. **(B)** MTT assay in GFP- or GFP-PAL31-overexpressed C6 after H_2_O_2_ treatment showing significant difference (cytoprotective effect of PAL31) at 0.5 mM and 1 mM H_2_O_2_ treatment between C6/GFP and C6/PAL31 groups. The data in each dosage were analyzed by two-way ANOVA and Bonferroni post hoc test. *P < 0.05, GFP (+H_2_O_2_) compared to PAL31 (+H_2_O_2_), n = 4, at 0.5 mM and 1 mM. **(C)** Western blot analysis of pal31 siRNA-treated C6 showing knockdown of PAL31 expression by PAL31 silencer using 41.5 (lane1), 83 (lane2), and 166 (lane 3) picomole of pal31 siRNA or 332 picomole negative control. Actin works as a loading control. **(D)** MTT assay in Negative- or PAL31 silencer transfected C6 after H_2_O_2_ treatment showing significant difference at 1 mM H_2_O_2_ treatment between C6/Negative and C6/PAL31 siRNA groups. *P < 0.05, Negative (+H_2_O_2_) compared to PAL31 siRNA (+H_2_O_2_), n = 4. Magnification 100X **(A)**.

To evaluate the cytoprotective effect of PAL31, H_2_O_2_ (0.5 ~ 1 mM) was added to GFP- or PAL31-transfected C6 for four hours. The survived cells were then analyzed by MTT assay. After H_2_O_2_ treatment, some cells were damaged and detached from the culture plates, only survived cells were able to reduce MTT into a blue formazan product. Figure 4A and B showed that H_2_O_2_ induced dose-dependent cell death in C6/GFP. Compared to H_2_O_2_-treated C6/GFP, C6/PAL31 were significantly more resistant to H_2_O_2_ -toxicity (P < 0.05 at 0.5 mM or 1 mM H_2_O_2_; Figure 4B). The survival ratio of C6/GFP by 0.5 mM H_2_O_2_ treatment was 78.52 ± 7.45% and that by 1 mM H_2_O_2_ treatment was 41.18 ± 6.13%. In C6/PAL31 group, the survival ratio by 0.5 mM H_2_O_2_ treatment was 101.32 ± 8.4% and that by 1 mM H_2_O_2_ treatment was 64.95 ± 10.45%.

To further confirm the function of PAL31, we employed pal31 siRNA to C6 culture. As shown in Figure 4C, the expression level of PAL31 protein was substantially reduced by pal31 siRNA at dose≧166 picomole/6 cm dish, compared with the negative control siRNA. To examine the knockdown cells’ response to toxin challenge, we subsequently treated cells with 1 mM H_2_O_2_ for 4 hours. Results demonstrated that pal31 siRNA treatment aggravated H_2_O_2_ toxicity to cells. These results consistently suggested that PAL31 might exert a cytoprotective effect on glial cells.

### Overexpression of PAL31 in C6 attenuated LPS/IFNγ-stimulated NO production

**Figure 5:**
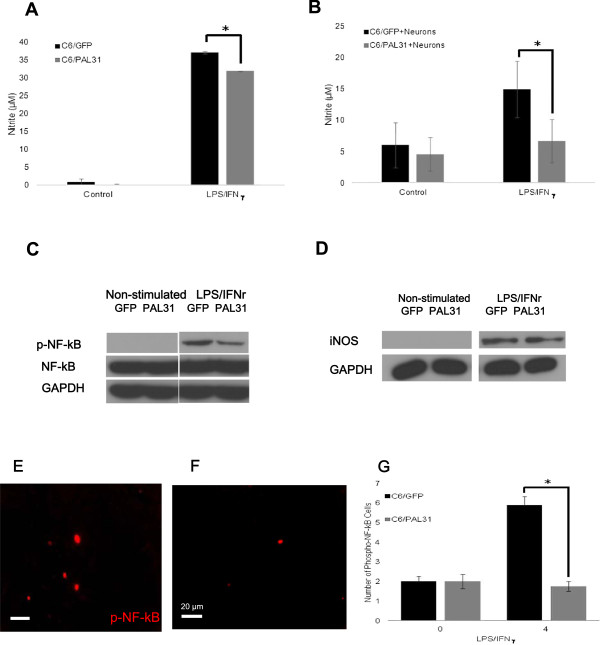
**Overexpression of PAL31 in C6 effectively attenuated LPS/IFNγ stimulation in C6 or in C6 after co-culture with spinal neuron-glial cultures. (A)** Level of nitrite release in C6 overexpressing GFP (C6/GFP) or GFP-tagged PAL31 (C6/PAL31) at 2 days after LPS/IFNγ stimulation **(B)** Levels of nitrite release in the transfected C6 co-cultured with spinal neuron-glial culture after LPS/IFNγ stimulation for 2 days. **(C)** Western blot analysis in C6/GFP or C6/PAL31 showing the expression of phosphorylated NF-kB is downregulated by PAL31 after LPS/IFNγ stimulation. NF-kB and GAPDH work as control. **(D)** Western blot analysis showing iNOS expression in the tranfected C6 co-cultured with spinal neuron-glial cultures after LPS/IFNγ stimulation. GAPDH works as a control. **(E)** Phosphorylated NF-kB-positive cells in LPS/IFNγ-stimulated neuron-C6 co-cultures (C6/GFP + neuron). **(F)** Phosphorylated NF-kB-positive cells in LPS/IFNγ-stimulated neuron-C6 co-cultures (C6/PAL31+ neuron). **(G)** Quantitative analysis of phosphorylated NF-kB positive cells in LPS/IFNγ-stimulated neuron-C6 co-culture shown in **(E&F)**. C6 glial cultures were transfected with pGFP or pPAL31 before seeding spinal cord neurons. *P < 0.05, C6/GFP (+LPS/IFNγ) compared to C6/PAL31(+LPS/IFNγ), n = 3 ~ 4.

The transfected C6 (C6/GFP or C6/PAL31) were stimulated by LPS and IFNγ, the culture medium were collected at 2 days later to evaluate NO production. As shown in Figure 5A, overexpressing PAL31 in C6 cells after LPS/IFNγ treatment produced 31.93 ± 0.08 μM nitrite which was significantly less nitrite level than C6/GFP-LPS/IFN γ treated group (37.13 ± 0.34 μM nitrite; P < 0.05). To investigate whether PAL31 overexpression suppressed NF-kB signaling on LPS/IFNγ stimulation, we treated C6/GFP and C6/PAL31 with LPS/IFNγ for shorter period (2 hr) and harvested cells for western blot analysis. As a result, Figure 5C shows a reduced level of phosphorylated NF-kB in LPS/IFNγ –treated C6/PAL31, compared to that in C6/GFP.

To confirm the overexpressed PAL31 in C6 glial cells may protect neurons from cell death by NO, we used the co-cultures assay (spinal cord neurons seeded to C6/GFP or C6/PAL31) and collected the conditioned medium to evaluate NO production. And cells were harvested for western blot or morphological analysis. Figure 5B showed that C6/GFP + Neurons (+LPS/IFNγ) group released 14.89 ± 4.5 μM nitrite to the conditioned medium. C6/PAL31 + Neurons group (+LPS/IFNγ) generated significantly less nitrite (6.63 ± 3.45 μM; P < 0.05) to the medium. Figure 5D demonstrates a reduced iNOS expression in PAL31-overexpressed cocultures than in GFP-cocultures after LPS/IFNγ treatment. The numbers of pNF-kB-positive cells in LPS/IFNγ-treated PAL31 cocultures (Figure 5F) was significantly less than that in LPS/IFNγ-treated GFP cocultures (Figure 5E). Figure 5G confirms that this response is related to NF-kB-mediated signaling. Combining these data in Figures 2, 3, 4 and 5, our *in vitro* model could mimic the *in vivo* model to prove that PAL31 in glia may have the important functions of immunomodulation after spinal cord injury.

## Discussion

The central observation of the present study is that PAL31 overexpression was found to localize in glia in addition to infiltrated macrophage/microglia in the injured spinal cord. This work examines the role of enhanced PAL31expression in spinal cord cultures, emphasizing on glial cells. Results showed that PAL31 overexpression in mixed glial cultures or in C6 astroglia was beneficial. This conclusion is supported by the following evidence. First, anti-inflammatory effect of overexpressing PAL31 was shown in primary mixed glia culture. Second, enhanced expression of PAL31 in C6 astroglia protected cells from H_2_O_2_ toxicity. By contrast, knockdown expression of PAL31 rendered C6 more susceptible to H_2_O_2_ toxicity. However, this did not affect the proliferative activity. Third, overexpression of PAL31 in C6 astroglia attenuated LPS/IFNγ-stimulated NO production. Lastly, inhibiting LPS/IFNγ stimulation by PAL31 was observed in glia or C6 after co-culture with neuronal cells. The primary co-culture study demonstrated our *in vitro* assay mimicking the *in vivo* model to evaluate the neuron-glial interaction during inflammation.

After LPS/IFNγ stimulation, nuclear factor κB (NF-κB) is phosphorylated, translocated into nucleus and activate transcription of downstream genes including iNOS [34,35]. In the present study, levels of NF-kB phosphorylation and nitrite were concurrently reduced by PAL31 overexpression in C6 and co-cultures after LPS/IFNγ stimulation. This might suggest that PAL31 suppress NF-kB signaling and thus reduce iNOS expression on LPS/IFNγ treatment.

The functions of glia after spinal cord injury include scar formation to prevent further neural damages [36] and protect neurons [37]. To further examine the functions of PAL31 in glial cells, we employed homogenous cell population-C6 astroglia which was generated from rat glioma and expressed S100 protein [38]. Furthermore, the transfection efficiency of GFP-tagged PAL31 in C6 was higher than in primary mixed glial culture. As shown in Figure 3B, the overexpressed PAL31 was specifically localized in the nucleus due to its nuclear localization signal in its protein sequence, consistent with previous studies [39,40]. Western blot analysis of these transfected C6 further confirms overexpression of GFP-tagged PAL31 with correct protein size and immunoreactivity (Figure 3C). PAL31 had been reported to co-localize with PCNA which participated in DNA replication and repair [16]. The cell cycle progression in S phase needs PAL31 [40]. However, the present results could not obtain similar effect. Overexpression of PLA31 did not alter PCNA level neither the proliferative activity in C6 cells (Figure 3C and D). Interestingly, PAL31 overexpressing in C6 could significantly reduce H_2_O_2_-induced cell death (Figure 4). This effect might be due to overexpressed PAL31 working as a caspase-3 substrate [17], rendering the PAL31-transfected cells more resistant to H_2_O_2_ toxicity. By contrast, PAL31 knockdown cells were more susceptible to H_2_O_2_ challenge. The results of over-expression and the small interfering RNA (siRNA) knockdown of PAL31 in C6 cells further suggested that the function of PAL31 might be negatively regulated.

Astrocytes become “reactive” in response to spinal cord injury. Nevertheless, the harmful and beneficial functions of reactive astrocytes are not well understood. The roles of astroglia in neural regeneration after spinal cord injury were still unclear. Immunomodulation of astroglia might play an important role. Previous studies demonstrated that iNOS upregulation in glia cells caused neurodegeneration in Parkinson’s disease [41] and the activated immune cell in brain were correlated with Alzheimer disease [42]. These studies gave us a hit to overcome the unfavorable microenvironment for regeneration after spinal cord injury. Modulation of astroglial reactivity should be paid more attention. In our previous studies of spinal cord regeneration, PAL31 was suggested to play a key role in spinal cord regeneration after injury [2]. The present findings are in keeping with our previous observations that both microglia and GFAP positive astroglia enhanced PAL31 expression near the lesion site after transected spinal cord injury (Figure 1). This phenomenon might be related to the important endogenous regenerative mechanisms. Reactive astrocytes exert both pro- and anti-inflammatory functions at different locations and at different times in response to injury and during repair. There were many studies focused on astroglia as a therapeutic target [43]. PAL31 effectively reduced LPS/IFNγ stimulation and also protected cells from H_2_O_2_ toxicity in astroglia in the present study. PAL31 may provide new means to study the activities and regulation of reactive astrocytes after CNS insults *in vivo*.

## Conclusions

In conclusion, the present study provides evidence that overexpressed PAL31 in glia cells could reduce LPS/IFNγ stimulation and H_2_O_2_ toxicity. Together with our previous study of immunomodulatory role of PAL31 in macrophages after SCI, this study suggests a beneficial role of PAL31 in glial cells after injury. A better understanding of glial cell roles during the complex multicellular interactions after CNS insults is important in developing potential therapeutic interventions.

## Competing interests

The authors declare that they have no competing interests.

## Authors’ contributions

FWT and DYL carried out cell culture and IHC studies. FWT and MJT participated in the design of the study and drafted the manuscript. MJT, WCH and HC conceived of the study, participated in its design and coordination, and collectively prepared the manuscript. All authors read and approved the final manuscript.

## Supplementary Material

Additional file 1 PAL31 knockdown clones showing the sequence of siRNA of PAL31. Click here for file
