# Supplementary material for: Cytoprotective and anti-inflammatory effects of PAL31 overexpression in glial cells
Source: J Biomed Sci. 2014 Jul 17;21(1):60. doi: 10.1186/1423-0127-21-60 (PMC4112974; doi:10.1186/1423-0127-21-60)
Supplement: Additional file 1 — PAL31 knockdown clones showing the sequence of siRNA of PAL31. [file 1423-0127-21-60-S1.pdf]

Supporting information

PAL31 knockdown clones showing the sequence of siRNA of PAL31.

Target Gene Symbol : Anp32b; Gene Alias (es) : PAL31

siRNA ID #: s139353	
Sense (5'->3')	GAACUUGUCUUGGACAAUUtt
Anti-sense	AAUUGUCCAAGACAAGUUCtt
siRNA ID #: s139354	
Sense (5'->3')	GAGUUCCUCAGUUUAAUAAtt
Anti-sense	UUAUUAAACUGAGGAACUCta
